# Archaeorhizomycetes Spatial Distribution in Soils Along Wide Elevational and Environmental Gradients Reveal Co-abundance Patterns With Other Fungal Saprobes and Potential Weathering Capacities

**DOI:** 10.3389/fmicb.2019.00656

**Published:** 2019-04-04

**Authors:** Eric Alejandro Pinto-Figueroa, Emily Seddon, Erika Yashiro, Aline Buri, Hélène Niculita-Hirzel, Jan Roelof van der Meer, Antoine Guisan

**Affiliations:** ^1^Department of Ecology and Evolution, University of Lausanne, Lausanne, Switzerland; ^2^Department of Fundamental Microbiology, University of Lausanne, Lausanne, Switzerland; ^3^Institute of Earth Surface Dynamics, University of Lausanne, Lausanne, Switzerland; ^4^Center for Primary Care and Public Health (Unisanté), University of Lausanne, Lausanne, Switzerland

**Keywords:** *Archaeorhizomyces*, fungi, Illumina sequencing, abiotic and biotic requirements, correlation analyses, network analysis, phyllosilicates, Swiss Alps

## Abstract

Archaeorhizomycetes, a widespread fungal class with a dominant presence in many soil environments, contains cryptic filamentous species forming plant-root associations whose role in terrestrial ecosystems remains unclear. Here, we apply a correlative approach to identify the abiotic and biotic environmental variables shaping the distribution of this fungal group. We used a DNA sequencing dataset containing Archaeorhizomycetes sequences and environmental variables from 103 sites, obtained through a random-stratified sampling in the Western Swiss Alps along a wide elevation gradient (>2,500 m). We observed that the relative abundance of Archaeorhizomycetes follows a “humped-shaped” curve. Fitted linear and quadratic generalized linear models revealed that both climatic (minimum temperature, precipitation sum, growing degree-days) and edaphic (carbon, hydrogen, organic carbon, aluminum oxide, and phyllosilicates) factors contribute to explaining the variation in Archaeorhizomycetes abundance. Furthermore, a network inference topology described significant co-abundance patterns between Archaeorhizomycetes and other saprotrophic and ectomycorrhizal fungal taxa. Overall, our results provide strong support to the hypothesis that Archaeorhizomycetes in this area have clear ecological requirements along wide, elevation-driven abiotic and biotic gradients. Additionally, correlations to soil redox parameters, particularly with phyllosilicates minerals, suggest Archaeorhizomycetes might be implied in biological rock weathering. Such soil taxa-environment studies along wide gradients are thus a useful complement to latitudinal field observations and culture-based approaches to uncover the ecological roles of cryptic soil organisms.

## Introduction

Microorganisms, like soil fungi, were recently shown to follow similar biogeographic patterns to macroorganisms, such as vascular plants, and were shown to be influenced by similar variables, such as climate and soil composition (Pellissier et al., 2014; Tedersoo et al., 2014). As for macroorganisms, their distributions can thus be expected to be, at least partly, driven by their environmental requirements (i.e., if speaking at the level of their constituent species, by their environmental niche).

As formalized by Hutchinson (Hutchinson, 1957), the realized environmental niche – or ecological niche (Martínez-Meyer et al., 2013) – describes the N-dimensional envelope of abiotic conditions, constrained by biotic interactions, which allow populations of a particular species to develop and persist (Pulliam, 2000; Wiens et al., 2010). Typically captured from field observations, the ecological niche concept has greatly facilitated our understanding of the distribution of species (Araújo and Guisan, 2006; Soberón and Nakamura, 2009). The same approach can be applied to higher taxonomic levels to assess the envelope of environmental requirements of all species within a group (Hadly et al., 2009; Smith et al., 2019), although in such case, the positive population growth criteria cannot be applied to the whole group.

Mountain regions with wide elevation-driven environmental gradients, in particular, provide a unique opportunity for studying environmental requirements of taxa because of the strong eco-physiological and abiotic variations observed within a confined geographic area (Becker et al., 2007; Pottier et al., 2013; Descombes et al., 2016). Elevational gradients also offer several features that make them suitable for microbial biogeographical studies: sampling strategies can be more easily based on stratifying environmental information (Hirzel and Guisan, 2002); steep changes over short distances allow reproducible field experiments (Pellissier et al., 2013); and the elevation forms a natural laboratory to track climate change and land use on species diversity (Becker et al., 2007). More recently, biogeographical studies have started focusing on the distribution of soil microorganisms along elevational gradients (Bryant et al., 2008; Fierer et al., 2011; Wang et al., 2012; Pellissier et al., 2014; Tedersoo et al., 2014; Yashiro et al., 2016), but so far very few have attempted to determine the environmental requirements of cryptic soil fungi as done here along wide continuous gradients.

Correlative studies revealing clear environmental determinism of mycorrhizal fungi at global (Tedersoo et al., 2014) or regional (Pellissier et al., 2014; Jarvis et al., 2015) scales strongly support that environmental requirements also govern the distribution and assemblage of soil fungi across latitudinal and elevational gradients. These studies describe edaphic factors, such as pH, phosphate, and nitrogen concentration (Pellissier et al., 2014; Shen et al., 2014; Rincón et al., 2015), and climatic variables, such as mean annual temperature or growing degree-days, as predictors of fungi distributions (Pellissier et al., 2013, 2014; Tedersoo et al., 2014). Other studies revealed the importance of biotic interactions for fungi, such as with plants (e.g., fungi as predictors of plants in Pellissier et al., 2013), but these studies have mostly been limited to mycorrhizal fungal species (e.g., van der Heijden et al., 1998, 2015; Tedersoo et al., 2010, 2012). Yet, most insights about the environmental requirements of fungi in nature have so far been restricted to contrasting a few different environmental conditions at a limited number of sites, often mainly contrasting different soil horizons (depth) or soil composition (e.g., Dickie et al., 2002; Dickie, 2007; Taylor et al., 2014), whereas several studies focused only on mycorrhizal fungi (e.g., Buée et al., 2007; Lekberg et al., 2007). These studies inform on the drivers of fungal communities in specific habitats and soil horizons, but do not provide a continuous and comprehensive view of fungal requirements (i.e., fitting continuous responses along environmental gradients) that account for multiple environmental factors (see Guisan et al., 2017). Although Hutchinson’s niche concept was originally defined – and should best be used – at the species level, the idea that taxonomic groups above and below the species level can also strive within a clear envelope of environmental conditions also led some authors to apply the niche concept to infra-specific (Pearman et al., 2010; Banta et al., 2012) or supra-specific (Hadly et al., 2009; Smith et al., 2019), levels. Although not formally corresponding to Hutchinson’s definition, the latter case can inform on the environmental requirements of an entire fungal group or clade (e.g., genus, family, order, or class). Advancing our understanding of the ecological requirements and spatial distributions of soil fungi at multiple taxonomic depth will require sampling soils across large regions spanning wide elevation and other abiotic and biotic gradients (e.g., Pellissier et al., 2014; Tedersoo et al., 2014 for soil fungi, Yashiro et al., 2016, 2018 for soil bacteria).

Archaeorhizomycetes comprise a deep branching phylogenetic (i.e., ancient) class of non-mycorrhizal and largely non-cultured fungi (but see Menkis et al., 2014) belonging to the subphylum Taphrinomycotina (Ascomycota) and so far only includes one described order, (Archaeorhizomycetales), one family (Archaeorhizomycetaceae), and one genus (*Archaeorhizomyces*) (Rosling et al., 2011), but the classification may still evolve given this lineage is still largely uncharacterized. It comprises hundreds of cryptically reproducing filamentous species without known fruiting structures, globally distributed in different world regions and in various terrestrial ecosystems (Porter et al., 2008; Rosling et al., 2011, 2013). These include soils of pacific coastal temperate conifer rainforest (Levy-Booth et al., 2018), cold temperate *Nothofagus* forests (Fernández-Martínez et al., 2017), of boreal forests (Clemmensen et al., 2015), and of arctic tundra (Borg Dahl et al., 2017), where Archaeorhizomycetes can locally dominate some soil compartments (e.g., in rhizosphere, Urbina et al., 2018; in arctic soils, Borg Dahl et al., 2017), but with overall high variations in relative abundances (e.g., along a deglaciation chronosequence; Fernández-Martínez et al., 2017). As first proposed by Rosling et al. (2011), together with the more detailed description of the type species *Archaeorhizomyces finlayi*, Archaeorhizomycetes are non-pathogenic plant root and rhizosphere associated fungi with putative saprotrophic activity (Rosling et al., 2011). The presence of sequence signatures has suggested Archaeorhizomycetes to occupy plant rhizospheres in deeper soil horizons with low pH and high nutrient turnover (Rosling et al., 2013; Clemmensen et al., 2015; Carrino-Kyker et al., 2016; Urbina et al., 2018). Archaeorhizomycetes spp. often dominate such sites and are commonly associated with mycorrhizal fungi (Choma et al., 2016). In addition, they seem to show specificity for certain plant types (e.g., *Tsuga*, *Picea*, and *Pinus* spp.; Rosling et al., 2013). Despite this wealth of information (Rosling et al., 2013), the absence of systematic sampling of fungal communities along environmental gradients makes the ecological role and trophic status of Archaeorhizomycetes in terrestrial ecosystems still elusive (Choma et al., 2016; Urbina et al., 2018). Since most Archaeorhizomycetes remain uncultured and their taxonomic diversity is poorly understood (Hughes Martiny et al., 2006), addressing their biogeography through molecular tools is cumbersome and, even if still prone to biases due to the used bioinformatic workflows (Rosling et al., 2013; Choma et al., 2016), large progress on their biogeographic determinants can still be expected through running a large and systematic metagenomics-based field survey.

The advance we bring here is to use a large soil metagenomics dataset sampled across wide environmental gradients in the Western Swiss Alps (Yashiro et al., 2016, 2018). The area was sampled in a random-stratified strategy aiming to cover a gradual change in abiotic (i.e., edaphic and climatic variables) and potentially biotic conditions. We mine this metagenomics DNA sample set specifically for Archaeorhizomycetes (indeed, here with only one genus, *Archaeorhizomyces*, included), in order to better assess the realized environmental requirements (“niche”) of this group in this area and along the abiotic gradients investigated, and its potential biotic relationships with plants and other fungi. We used a statistical approach to quantify those abiotic and biotic factors correlated to Archaeorhizomycetes abundance, and this way identify the envelope of suitable environmental conditions for this group (and the currently single order, family and genus included; thus overall at a supra-specific level; Hadly et al., 2009; Smith et al., 2019). More specifically, we aimed to answer the following three questions: (i) Do Archaeorhizomycetes (and here at least the single *Archaeorhizomyces* genus it includes) exhibit geographic patterns along environmental – and especially elevational – gradients? (ii) Are there abiotic factors that significantly explain the observed patterns? (iii) Are there significant correlations between Archaeorhizomycetes marker sequence abundances and that of other organisms such as plant species and fungi?

## Materials and Methods

### Analytical Framework

We used a large environmental DNA sample set and environmental variables from 103 sites obtained by a random-stratified sampling across wide spatial and elevational gradients in the Western Swiss Alps during summer 2012 (July–September). Fungal marker sequences including Archaeorhizomycetes were amplified from the purified environmental DNAs, sequenced and analyzed to operational taxonomic units (OTUs) as described below. Those abiotic and biotic variables with the greatest correlation with Archaeorhizomycetes OTUs were selected using Spearman rank correlations. Next, we fitted generalized linear models (GLMs) with the abundance of Archaeorhizomycetes sequences as the response variable and the selected environmental variables as predictors to identify the significant abiotic variables explaining this whole group’s “ecological niche.” Lastly, we analyzed the correlation of Archaeorhizomycetes with a set of biotic variables, using network inference analyses, to identify significant biotic relationships of Archaeorhizomycetes with other fungal groups, including also all the previously identified significant abiotic variables.

### Study Area and Data Collection

**Figure 1:**
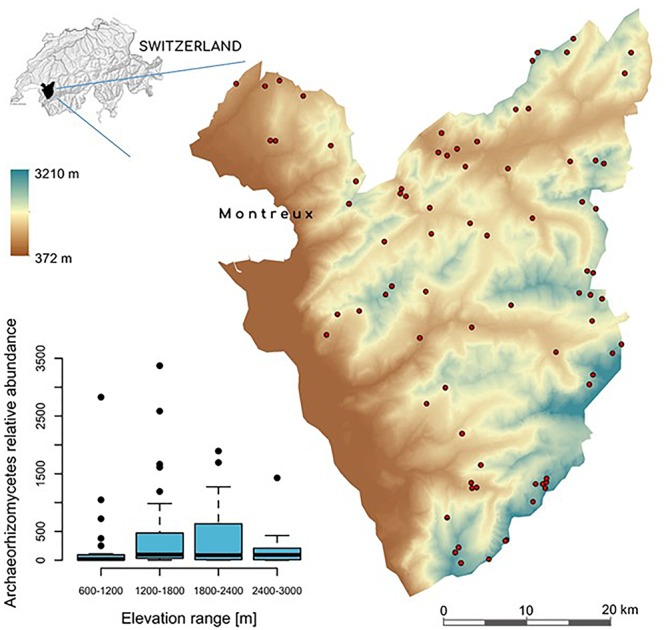
Digital elevation model (DEM) of the study area in the Western Swiss Alps (700 km^2^). The small map on the top left shows the location of the study area, colored in black, within Switzerland. Red dots correspond to the 103 sampling sites across the >2,500 m-wide elevation gradient. The boxplot on the bottom left, represents the relative abundance of Archaeorhizomycetes OTUs across all samples, categorized in elevational bins.

This study was performed in the Western Swiss Alps, across an area of ca. 700 km^2^ spanning an elevation gradient from 374 to 3210 m (Figure 1). This area had already been extensively investigated to understand plant and soil bacteria distributions along an elevation gradient (Dubuis et al., 2011, 2013; Ndiribe et al., 2013; Pottier et al., 2013; Yashiro et al., 2016, 2018; Descombes et al., 2017)^1^. Soil samples were collected from 103 plots of 4 m^2^ of open (non-forested) vegetation between 800 and 3000 m in elevation, as described previously (Yashiro et al., 2016, 2018) using a balanced random-stratified sampling design (Hirzel and Guisan, 2002) based on elevation, slope, and aspect. Forest areas were excluded to allow a better comparison of fungal composition in grassland-like habitats along the full elevation gradient. From each sampling site, we measured a total of 49 local edaphic factors (for details on the specific methods, see Buri, 2014; Yashiro et al., 2016; Buri et al., 2017), and 7 climatic variables at 25 m resolution (Descombes et al., 2017; Supplementary Table S1). The percent plant cover for 448 plant species across all plots was inventoried as previously described (Dubuis et al., 2011, 2013).

### Molecular Analyses

DNA samples had been extracted and purified from mixed and sieved soil cores (five per site), as described previously (Yashiro et al., 2016). To address fungal diversity, we exploited sequence variation in the fungal internal transcribed spacer region (ITS1), as proposed by Schmidt et al. (2013). ITS1-regions were amplified with primers ITS1F, 5′-GAACCWGCGGARGGATC-3′ (Schmidt et al., 2013) and ITS2, 5′-GCTGCGTTCTTCATCGATGC-3′ (White et al., 1990). Both ITS-primers were coupled at the 5′ end to specific sets of barcode sequences to allow sample multiplexing. *In silico* tests to evaluate coverage (Supplementary Figure S1) and details of the used ITS-primer barcode couples are provided in the Supplementary Tables S2, S3.

PCR amplifications were carried out in a total volume of 50 μl containing 21 μl H_2_O, 10 μl of 5×-concentrated PrimerSTAR buffer (Takara, Shiga, Japan), 10 μl of 5 M Betaine solution, 4 μl of a (10 mM mixture of all four deoxy-nucleotides, 0.6 μl of PrimerSTAR HS DNA polymerase (Takara, Shiga, Japan), 2 μl barcoded primer-pair (final concentration 0.2 μM), and 2 μl DNA sample (DNA elution, 25-folds). PCRs were performed on a GeneAmp model 9700 (Applied Biosystems, Foster City, CA, United States) with the following conditions: initial denaturation at 98°C for 2 min, followed by 30 cycles of each, 98°C for 1 min, 57°C for 5 s, and 72°C for 40 s, and a final extension at 72°C for 5 min. The obtained amplicons were purified by using a QIAquick PCR Purification kit (Qiagen, CA, United States) and eluted in 20 μl of elution buffer (Qiagen, CA, United States). Purified PCR products were quantified using the Quant-IT assay kit (Life Technologies, Carlsbad, CA, United States), and pooled to approximate equimolar amounts. Libraries were prepared with the TruSeq DNA sample preparation kit (Illumina, San Diego, CA, United States) for paired-end sequencing (2 × 101 nt) (to see the overall PCR strategy, Supplementary Figure S2). Sequencing was carried out on an Illumina HiSeq 2500 platform at the Genomic Technologies Facility of the University of Lausanne.

### Archaeorhizomycetes and Fungal OTUs

Raw fastq reads were demultiplexed and quality filtered according to both the Illumina adapter and the specific paired-barcodes coupled to the ITS primers. Trimming, removal of short reads and initial removal of anomalous reads were done using custom Bash scripts (Yashiro et al., 2016). Forward and reverse paired-end reads were then trimmed to 92 nt, concatenated, and prepared for QIIME 1.8 (Caporaso et al., 2010). A reference dataset was created from the fungal ITS QIIME/UNITE dataset (version 12.11). This dataset contains non-redundant fungal ITS1 sequences trimmed and concatenated in the same way as the paired-end fastq reads (Supplementary Figure S2). Given that only the outer regions of the ITS1 were used for taxonomy assignment, our method does not bias against fungi with very long ITS1 regions. To evaluate the effect of trimming and concatenation in shorter ITS1 regions, such as those of Archaeorhizomycetes, we compared the ITS1 region length between the complete and the concatenated Archaeorhizomycetes sequences obtained from the dataset (UNITE-QIIME 12.11) (Supplementary Figure S3). Sequences between 183 and 192 bp were obtained (20 bp of the 18S, 50 bp of the 5.8S region and a variable region of 112 bp for *Archaeorhizomyces finlayi* (Schoch et al., 2014) or 121 bp for *Archaeorhizomyces borealis* (Menkis et al., 2014). Consequently, the concatenated sequence of 184 bp is not expected to present a sufficient overlap in the middle sequence to be detected with a software.

High quality paired-end reads were processed using open-reference OTU picking (Rideout et al., 2014), a hybrid method that implements both closed-reference and *de novo* clustering using the UCLUST algorithm (Edgar et al., 2011). Referenced and *de novo* OTUs were combined into a single OTU table (for more details on the overall strategy of fungal metabarcoding using HiSeq Illumina, see Supplementary Figure S4). OTU clusters with less than ten reads were removed (Pagni et al., 2013; Schmidt et al., 2013). To account for differences in sequencing per site, all samples were rarefied (Gotelli and Chao, 2013). Because *de novo* OTUs for fungi clusters were not very different to referenced OTUs as shown by Procrustes analyses (see Thioulouse et al., 2018) in QIIME 1.8 (Caporaso et al., 2010; Supplementary Figure S5), and because they are not currently well defined (Westcott and Schloss, 2015), all the subsequent analyses were done using the relative abundance of referenced OTUs.

### Preselection of Biotic and Abiotic Variables

To use a reasonable number of explanatory variables in the analyses (typically a 1:10 ratio between variables and observations), an initial filtering step was performed using Spearman rank correlations (Clarke and Ainsworth, 1993) to select the biotic and abiotic variables most correlated with the total relative abundance of Archaeorhizomycetes reads. The selection was performed separately within two groups of explanatory variables: biotic (222 fungal OTUs at genus level; 448 plant species) and edaphic (49 variables). No functional fungal group was considered here as defining them is still difficult (Frac et al., 2018). In addition, seven climatic variables already used in previous studies (e.g., Pellissier et al., 2013; Yashiro et al., 2016; Supplementary Table S4) were then added. The three sets of selected variables were jointly visualized by a co-abundance matrix using *ggplot2* in R (Wickham, 2009).

### GLM Analyses on Abiotic Variables

To identify significant abiotic (i.e., edaphic and climatic) variables explaining Archaeorhizomycetes’ abundance, linear and second order polynomial functions were fitted with GLMs in R (Version 3.2.1, R Development Core Team, 2015) using a Gaussian distribution and identity link function (McCullagh and Nelder, 1989). After analyzing residuals versus predicted values (Supplementary Figures S6A, S7A), and testing the assumptions of normality (Nelder and Wedderburn, 1972; Supplementary Figures S6B, S7B), GLMs were fitted on the ten edaphic and seven climatic variables with untransformed and log-transformed Archaeorhizomycetes abundances (Supplementary Figure S8). A backward stepwise approach based on the Akaike information criterion (AIC) (Burnham and Anderson, 2004) was used to retain the final variables in the multiple regression models, choosing the model with the smallest AIC. Values were corrected for multiple comparisons using the Benjamini-Hochberg method (Benjamini and Hochberg, 1995).

### Network Analyses

A co-abundance network inference analysis between Archaeorhizomycetes, the 12 selected biotic variables (i.e., fungal OTUs and plant species) and the abiotic variables selected from the previous correlation and GLM analyses was performed using the SparCC Python module based on Spearman correlations (Friedman and Alm, 2012). To measure the significance of the predicted relationships, pseudo *p*-values defined as the percentage of times a correlation at least as extreme as the observed were obtained by bootstrapping (1000 resampled datasets) (Friedman and Alm, 2012) were used. To convert the pseudo *p*-values into significance values, pseudo *p*-values of near zero were converted to a large positive value by multiplying by 1 × 10^-8^ and applying a logarithm. Positive converted values >1 were considered significant. To visualize the *p*-values in the network, we used the *igraph* R package (Csárdi and Nepusz, 2006).

## Results

### Fungal OTUs and Archaeorhizomycetes Abundance

A total of 46.9 million high-quality paired-end reads across all sites were obtained. Open-reference clustering using a threshold of 97% identity indicated a high variation of the number of reads per site (Supplementary Table S5). Rarefaction curves at 21,500 reads per site showed asymptotic or nearly asymptotic behavior for most sites (Supplementary Figure S9), suggesting that most of the fungal diversity was captured. From a total of 2.2 million rarefied reads, only 2,094 OTUs (37.48%) could be reference-clustered, whereas 3,497 OTUs (62.52%) were *de novo* clustered (Supplementary Figure S10). Among the referenced sequences, 36,635 ITS1 sequences were classified as Archaeorhizomycetes (*Archaeorhizomyces*). This number corresponded to 13.3% of total fungal OTUs (i.e., 278 OTUs) assigned after rarefaction. Archaeorhizomycetes OTUs mostly belonged to four groups of non-cultured Archaeorhizomycetes, represented by GenBank accession numbers DQ182455, GU174301, GU174341, and GU174343 (Rosling et al., 2011). Ranking abundance curve showed that Archaeorhizomycetes was the most abundant and prevalent across sites (Supplementary Figure S11). Relative abundance of Archaeorhizomycetes varied considerably (min = 0; median = 78; mean = 378; max = 3372) and displayed a significant “humped-shape” curve as a function of elevation, characterized by highest abundances at middle elevations (Figure 1).

### Preselection of Abiotic and Biotic Variables

**Figure 2:**
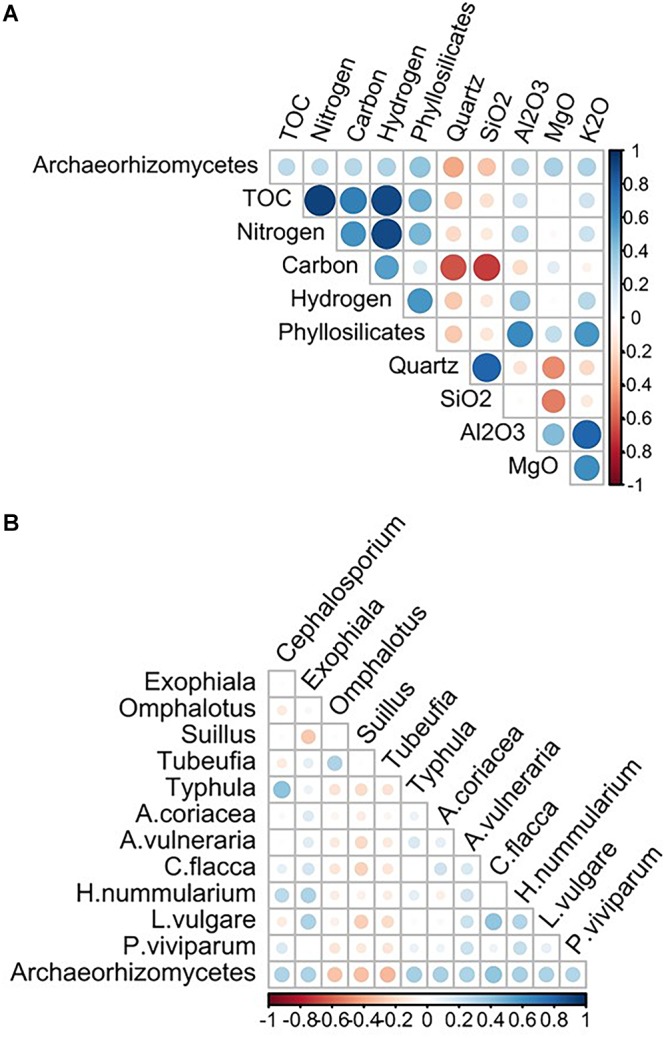
Archaeorhizomycetes OTU relative abundance correlations with 10 edaphic factors **(A)** abiotic and 12 biotic variables **(B)**, of which 6 fungal genera and 6 plant species. Correlations are described as Spearman correlations, with positive interactions in blue, and negative in red. The shading color and size of the circles correspond to the strength of the correlation, on an absolute scale between 0 and 1.

Based on the Spearman correlation analyses, 12 biotic variables (six fungal OTUs and six plant species; Supplementary Tables S6, S7, respectively) and ten abiotic edaphic factors (Supplementary Table S8) were selected and used with the seven abiotic topo-climatic predictors in further fungi-environment analyses. Among abiotic edaphic factors, the abundance of Archaeorhizomycetes was most positively correlated with total organic compounds (organic material, carbon, nitrogen, and hydrogen), mineral components [magnesium oxide (MgO), potassium oxide (K_2_O), aluminum oxide (Al_2_O_3_)] and parent material (phyllosilicates) and negatively correlated with quartz content (Figure 2A). Regarding the biotic variables, OTUs of the fungal genera *Cephalosporium* (taxon synonymous *Acremonium*), *Exophiala* and *Typhula* were the most positively correlated with Archaeorhizomycetes OTUs, whereas those of *Omphalotus*, *Suillus*, and *Tubeufia* were the most negatively correlated with Archaeorhizomycetes (Figure 2B). Archaeorhizomycetes OTU abundance was most positively correlated with the plant species *Carex flacca*, *Helianthemum nummularium*, *Alchemilla coriacea*, *Anthyllis vulneraria*, *Leucanthemum vulgare*, and *Polygonum viviparum* (listed in order of highest to lowest correlation strength; Figure 2B and Supplementary Table S7).

### Archaeorhizomycetes’ Abiotic Environmental Requirements

**Figure 3:**
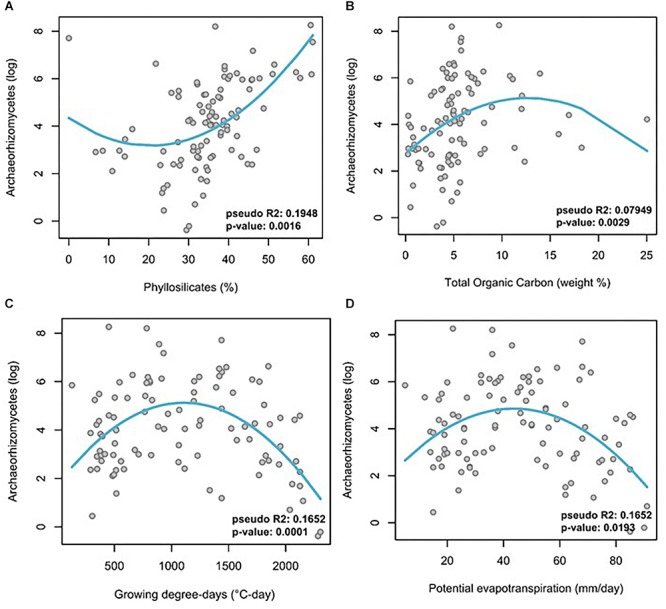
Log-transformed relative abundance of Archaeorhizomycetes OTUs across all samples as a function of abiotic factors. **(A)** phyllosilicates, **(B)** total organic carbon, **(C)** growing degree-days, and **(D)** potential evapotranspiration. Trend curves were calculated by mean of a univariate GLM using Gaussian family and identity link function. Note the unimodal “humped-shaped” curves for three of the abiotic factors, and a reverse unimodal curve for phyllosilicates.

Multivariate GLMs were obtained between observed Archaeorhizomycetes OTU abundance as the response variable and different edaphic and climatic variables as explanatory variables (Table 1, full models in Supplementary Table S9). After stepwise selection and Benjamini-Hochberg correction for multiple testing, the GLM analysis revealed that Archaeorhizomycetes relative abundance in the soil was most significantly explained by evapotranspiration, growing degree-days, monthly sum precipitation and minimum temperature for the climatic predictors (Table 1), as well as by total organic carbon, carbon, nitrogen, hydrogen, phyllosilicates and aluminum oxide for the edaphic predictors (Table 1). The goodness-of-fit (adjusted pseudo *R*^2^) of the model with these selected abiotic factors was 0.58. Inspection of univariate response curves in GLMs further revealed a linear increase of Archaeorhizomycetes relative abundance with phyllosilicate concentrations (Figure 3A), and “humped-shaped” dependencies with total organic carbon, growing degree-days and evapotranspiration (Figure 3B–D, respectively).

**Table 1:** Generalized linear model (GLM) and generalized quadratic model most significant results of Archaeorhizomycetes and edaphic and climatic factors.

Type	Factor	Estimate	std. error	*t*-Value	*p*-Value	BH
Edaphic	Nitrogen	-15.8727	4.768	-3.329	0.0014**	0.0041
	Hydrogen	8.2439	2.6333	3.1306	0.0025**	0.006
	Phyllosilicates	-0.1663	0.0632	-2.6302	0.0104*	0.0208
	Al_2_O_3_	0.8353	0.3383	2.4694	0.0158*	0.0272
	TOC^2^	-0.0818	0.0266	-3.0769	0.0029**	0.0064
	Nitrogen^2^	10.3445	3.089	3.3488	0.0013	0.0041
	Carbon^2^	0.1119	0.0312	3.5885	0.0006***	0.0026
	Hydrogen^2^	-3.2331	0.9003	-3.5913	0.0006***	0.0026
	Phyllosilicates^2^	0.0033	0.001	3.2703	0.0016**	0.0044
Climatic	gdd	0.018	0.0051	3.5588	0.0007***	0.0026
	psum	-0.037	0.0088	-4.2105	0.0001***	0.0012
	etp	-0.2312	0.0966	-2.3925	0.0193*	0.0308
	tmin^2^	0	0	2.5483	0.0129*	0.0238
	gdd^2^	0	0	-4.1216	0.0001***	0.0012
	psum^2^	0	0	3.6823	0.0004***	0.0026

*Generalized quadratic model results are indicated with a squared superscript. Levels of significance were evaluated by Benjamini-Hochberg (BH) multiple correction (^∗^*p*-value <0.05, ^∗∗^*p*-value <0.01, and ^∗∗∗^*p*-value <0.001). For details on: climatic variables, see Supplementary Table S4; Full list of GLMs results, see Supplementary Table S9. TOC, total organic carbon; Al_2_O_3_, aluminum oxide; gdd, growing degree-days; psum, sum precipitation days/growing season; etp, potential evapotranspiration; tmin, minimum monthly average temperature*.

### Global Abiotic and Biotic Network Inference

**Figure 4:**
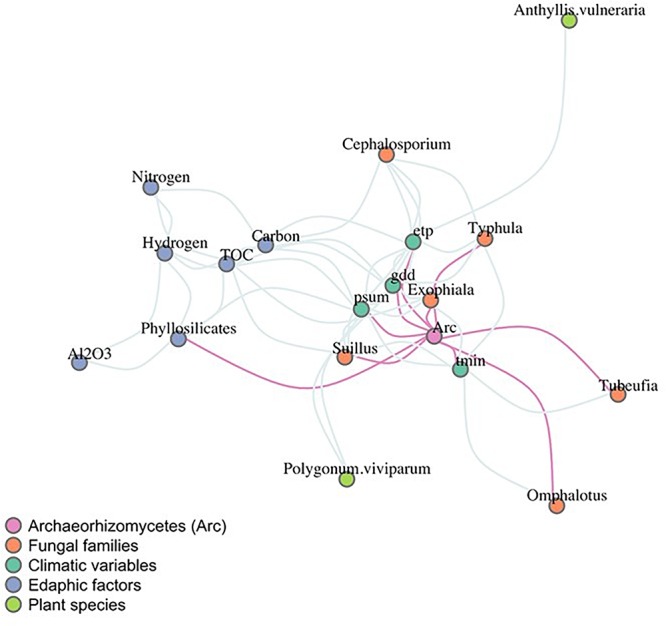
Inference network of co-occurrences between Archaeorhizomycetes, biotic, climatic, and abiotic variables. Nodes are colored according to the variable type as indicated in the color legend. Direct connections to Archaeorhizomycetes in pink represent the significant predicted relationships using the pseudo *p*-values. Tmin, minimum monthly average temperature; gdd, growing degree-days; etp, potential evapotranspiration; TOC, total organic carbon; psum, sum precipitation days/growing season.

Once the most explanatory abiotic factors were identified from the GLMs, correlation patterns were further explored using network inference (Figure 4). The network object consisted of 23 nodes and 75 significant edges (an average clustering coefficient of 0.46). Edaphic and climatic variables clustered together (see blue and dark green nodes, Figure 4). The Archaeorhizomycetes environmental edges (in pink) associated significantly with minimum temperature, evapotranspiration, growing degree-days and monthly sum of precipitation (Figure 4). Once the biotic variables were added, phyllosilicate concentrations remained the unique significant edaphic factor directly associated with variations in Archaeorhizomycetes abundances (Figure 4). Regarding the biotic variables, Archaeorhizomycetes abundances correlated significantly with the fungal plant pathogen *Typhula* (GenBank AF193350), the saprotrophic fungus *Exophiala* (GenBank AB488490), the *Suillus* ectomycorrhizal fungal families (GenBank GU187544, GU553371, L54082, L54112), and the genera *Tubeufia* (GenBank AY916461) and *Omphalotus* (GenBank AY313271), both involved in vegetation decaying processes (Figure 4, details on the fungi detected are in Supplementary Table S6). None of the plant species considered had a significant direct correlation with Archaeorhizomycetes.

## Discussion

Soil fungi play fundamental roles in the functioning of terrestrial ecosystems (van der Heijden et al., 2008). Yet, the distribution and ecological role of most cryptic fungi are currently still under-determined (Tedersoo et al., 2014; Choma et al., 2016), limiting a more comprehensive understanding of natural soil ecosystems. The ancient fungal class Archaeorhizomycetes, one of the most ubiquitous and abundant cryptic groups of soil fungi, is described to have root endophytic properties and saprotrophic potential in the lab (Rosling et al., 2011). However, their precise environmental requirements and their ecological role in natural ecosystems remained largely unexplored. Here, we used a multivariate framework to explore and quantify the abiotic and biotic environmental requirements of the fungal class of Archaeorhizomycetes, and at least of the single *Archaeorhizomyces* genus it contains.

Our study revealed three major findings. First, Archaeorhizomycetes abundances are not evenly distributed across the elevation gradient, and thus in geographic space as well, but rather follow a “humped-shaped” relationship along elevation (Figure 1). Second, the environmental requirements of Archaeorhizomycetes are defined by different climatic (notably: minimum temperature, evapotranspiration, growing degree-days and sum precipitation) and edaphic factors (in particular: carbon, nitrogen, hydrogen, total organic carbon, aluminum oxide and phyllosilicates) (Table 1 and Supplementary Table S9). Third, network inference further revealed significant correlations between Archaeorhizomycetes, fungal saprophytes belonging to the genera *Typhula* and *Exophiala*, and the ectomycorrhizal *Suillus*. Overall, these results strongly support our hypothesis that the spatial variation in Archaeorhizomycetes abundance is at least partly driven by environmental determinism along wide abiotic gradients and by positive or negative associations with other fungal taxa. Unexpectedly, significant correlations to soil redox parameters, in particular with phyllosilicates minerals, suggest a potential involvement of Archaeorhizomycetes in rocks’ biological weathering. This would, however, require *in vitro* experiments (e.g., as suggested in Rosling et al., 2009) to be formally demonstrated.

Archaeorhizomycetes represented around 13% of the total referenced OTUs across the alpine landscape. This suggests that Archaeorhizomycetes are indeed important members of diverse terrestrial ecosystems, as concluded from previous studies (Rosling et al., 2011, 2013; Menkis et al., 2014). The maximum relative abundance of Archaeorhizomycetes among the sites sampled here was found at a mid to high elevations (1800–2400 m), suggesting that fungi in this group prefer environmental conditions found in subalpine and alpine vegetation belts. Although Archaeorhizomycetes have an apparent wide distribution (Rosling et al., 2013), our data thus indicate that particular environmental preferences can still be identified. From our sampling of open-habitats only, our results confirm that Archaeorhizomycetes accommodate well in treeless soil conditions with low temperatures, as previously shown for tundra biomes (Schadt et al., 2003; Borg Dahl et al., 2017). However, as Archaeorhizomycetes were also observed in boreal (Clemmensen et al., 2015) and cold *Nothofagus* forests (Fernández-Martínez et al., 2017), additional sampling within forests should be conducted within our study area to assess whether they also colonize forest soils and in which specific forest types.

Different environmental variables contributed to explain part of the variation in the relative abundance of Archaeorhizomycetes, and thus to define their environmental requirements in this region along the abiotic gradients considered. Notably, the climatic variables *growing degree-days*, *minimum temperature*, and *precipitation* correlated strongly with observed Archaeorhizomycetes abundances, suggesting that, in our open mountain habitats, they prefer colder and more humid conditions (Table 1 and Figure 3D). Our finding is in accordance with previous descriptions of Archaeorhizomycetes as being spring seasonal in tundra environments (Schadt et al., 2003). Borg Dahl et al. (2017) also showed Archaeorhizomycetes in cold tundra environments to be affected by shading and warming. A positive correlation with *total organic carbon* suggests that Archaeorhizomycetes proliferate at a higher carbon content in the soil, and thus, potentially depend on surrounding organic carbon sources, as had been proposed initially (Rosling et al., 2011). Other studies confirmed their dominance in the rhizosphere with putatively higher nutrient turnovers and deeper soils (Clemmensen et al., 2015; Fernández-Martínez et al., 2017), as well as in soils with lower pH and more available phosphorus (Carrino-Kyker et al., 2016). Further notable significant associations with edaphic factors *aluminum oxide* (Al_2_O_3_), *hydrogen*, and particularly *phyllosilicates* suggest that Archaeorhizomycetes may play a role in biological weathering (although this has not yet been shown experimentally for this group; see, e.g., weathering reactions in Box 1 in Hoffland et al., 2004). The extent of fungal biological rock weathering has been increasingly studied (Landeweert et al., 2001; Hoffland et al., 2004; Gadd, 2007; Shashank et al., 2016), with different fungi having been described as excreting organic acids, phenolic compounds, or siderophores that dissolve minerals (Hoffland et al., 2004). Trees may further enhance the activity of fungi for mineral weathering (Quirk et al., 2012), but our samples excluded sites with trees and our data therefore cannot confirm or refute possible Archaeorhizomycetes associations with trees and enhanced weathering. This would be a suitable direction for future research in our area.

Significant positive, as well as negative correlations, between Archaeorhizomycetes’ and other fungal abundances were inferred from network analysis (Figure 2B, 4). One of the positively associated fungi, *Typhula*, consists of psychrophilic soil fungi with saprophytic and pathogenic activity on dormant plant hosts during winter (Chang et al., 2006). Other co-occurring fungi are *Exophiala* species, also described as having saprotrophic and potential pathogenic activity (Barnes et al., 2016). However, negatively correlated fungi, such as the *Tubeufia* and *Omphalotus* genera, also include saprotrophic species (Fukasawa et al., 2010; Chaiwan et al., 2017). Archaeorhizomycetes were also negatively correlated to *Suillus* spp., described as ectomycorrhizal (Jarvis et al., 2015) and implicated in weathering processes (Hoffland et al., 2004; for more details on the correlated fungi see Supplementary Table S6). These correlations with other fungal taxa suggest that Archaeorhizomycetes might benefit in some cases from carbohydrates or nutrients released from organic matter according to substrate-dependent vertical partitioning of the resources across soil horizons. As different fungal species were shown to have coinciding fundamental niches, their realized niches could differ with respect to competition for nutrients (Gadgil and Gadgil, 1971; Bending, 2003; Lekberg et al., 2007; Bödeker et al., 2016) and lead them to occupy different soil depths (i.e., “niche” partitioning). This idea could also be tested in our area in further experimental approaches of combined consumption of organic matter by saprotrophic and ectomycorrhizal fungi, as suggested by Bödeker et al. (2016). We did not find any significant correlations between plant and Archaeorhizomycetes distributions, suggesting that they might not have specific root-associations as specific plant colonizers, at least not across the range of open grassland-type habitats investigated here. This is in contrast to studies describing potential associations of Archaeorhizomycetes with plant growth (Schadt et al., 2003) and thus would need to be further investigated. An interesting avenue to further explore could be whether distinct plants in different vegetation states along a successional gradient (e.g., from pioneer to late successional) have active root associations with Archaeorhizomycetes.

Although an increasing number of studies have proposed MiSeq Illumina for fungi in recent years (Schmidt et al., 2013; Bálint et al., 2014), at the time of our study we only had access to HiSeq Illumina 2500. Even though this technology is recognized for its great depth coverage (Taberlet et al., 2012), at that time it only allowed amplification of 100 bases forward and 100 bases reverse (although this has recently improved significantly). To counter that and to reference as many fungal sequences as possible, we decided to adapt the fungal reference dataset UNITE 12.11 and to use a hybrid method which generates both referenced and *de novo* OTUs (for more details about the strategy, see Supplementary Figures S1–S3). We recognize the high read recovery and later low usability of the HiSeq Illumina reads, in our case from 46.9 million high-quality reads, we used only 2.2 million after rarefaction (i.e., 20-fold factor). This data reduction came from the experimental decision to include in the rarefaction procedure (Gotelli and Chao, 2013) the sampling sites with very low sequencing reads (Supplementary Table S5). Despite this, rarefaction curves at 21,500 sequences showed that we managed to cover most of the fungal diversity across the study area (Supplementary Figure S9), as also shown by the Procrustes analysis plots comparing the non-metric multidimensional scaling (NMDS) topologies from *de novo* and referenced OTUs (Supplementary Figure S5). Here, we observed that *de novo* and referenced OTU picking correlate in almost 97% of cases, suggesting that *de novo* OTU clustering is highly dependent on the reference dataset (Rideout et al., 2014) and technically a pseudo *de novo* clustering strategy. For this reason, our analyses were performed only on the referenced OTUs. At the same time, we acknowledge that we may have underexplored the fungal diversity, which could have altered specific observations and conclusions made in this study. Indeed, the trimming of HiSeq sequences to 92 nt and their concatenation (184 nt overall), might have affected the OTU identification and/or underestimated the overall abundance of ITS1 regions shorter than 184 nt. Nevertheless, the length of the concatenated sequence is long enough to cover that of described ITS1 Archaeorhizomycetes sequence – *A. borealis* (192 nt; Menkis et al., 2014) and *A. finlayi* (183 nt; Schoch et al., 2014). This is supported by the findings of Tedersoo et al. (2015) who described a similar length for the variable region of ITS1 Archaeorhizomycetes. The representability of our dataset at Archaeorhizomycetes family level is supported by the diversity of Archaeorhizomycetes OTUs detected (*n* = 16).

We also acknowledge that the environmental envelope approach used here could be criticized for being applied to a whole fungal group. Based on current taxonomic knowledge, the Archaeorhizomycetes class includes so far only one genus *Archaeorhizomyces* (within a single family and single order; Rosling et al., 2011) and thus the approach used here can at least be considered reasonable at the genus level, where well-defined environmental requirements can already be identified in some cases (e.g., for plant, *Androsace* species being specialized in cold high-altitude or high-latitude climates; Boucher et al., 2012). Furthermore, environmental envelope studies are also found at coarser taxonomic levels (Smith et al., 2019), when for instance assessing niche conservatism across phylogenetic trees (Hadly et al., 2009). Another potential limitation when using environmental envelope approaches is the selection of the proper environmental variables in the model. Previous knowledge on the taxa is traditionally used to direct precise analyses (Austin, 2007; Mod et al., 2016; Petitpierre et al., 2017), but this information was absent in our study. Our idea instead was to reduce complexity by using pairwise Spearman rank correlations (Dormann et al., 2013). A limitation present in other studies is the use of presence-only or presence-absence data, rather than abundances, for quantifying and modeling environmental requirements and distributions of various organisms (e.g., plants, vertebrates and insects; Pellissier et al., 2012; Dubuis et al., 2013; Maiorano et al., 2013). It was, however, a strength of our study to use relative abundance from sequencing datasets directly, rather than transforming them into presence-absence data, which could have resulted in a loss of explanatory or predictive power. Still, environmental niche theory for microorganisms and the technical framework to use OTU relative abundance data in analyses and modeling are still in the early stages of development and would benefit from further improvements.

Finally, although our study identified several potential abiotic and biotic preferences of Archaeorhizomycetes (and here mainly of the *Archaeorhizomyces* genus), further support to these conclusions should come from experimental approaches (as, e.g., in Fukami and Morin, 2003, see Lee-Yaw et al., 2016), which could be optimally designed along environmental gradients based on our models and results. Likewise, our study shows the usefulness of large-scale DNA sets and next-generation amplicon sequencing as a template framework to uncover the ecological roles and environmental requirements of other cryptic soil organisms along wide natural environmental gradients, which for many are still uncharacterized (Choma et al., 2016; Hibbett, 2016; Peay et al., 2016). This is still of high importance because, despite significant advances in recent years regarding the descriptions of environmental niches for macroorganisms, still much remains to be known about the environmental requirements and niches of microorganisms, opening promising avenues for future research.

## Author Contributions

AG, JvdM, and HN-H designed the initial soil metagenomics project in the Swiss Alps. EP-F, EY, AG, HN-H, and JvdM designed the initial sampling survey. EP-F and EY conceived the soil DNA sampling protocole, with AB for the soil physical properties. EP-F, ES, AG, and JvdM designed the specific analyses for this study. AB conducted all soil analyses. EP-F and EY performed the DNA extraction and ITS fungal Illumina pre-processing. EP-F performed the sequence data analyses for the determination of fungal OTUs. EP-F performed the correlative analyses and wrote the initial manuscript. All authors contributed to the interpretation of results and further refinements of the manuscript.

## Conflict of Interest Statement

The authors declare that the research was conducted in the absence of any commercial or financial relationships that could be construed as a potential conflict of interest.

## Footnotes

1 http://rechalp.unil.ch

## Supplementary Material

All embedded in one single document (Data Sheet 1.pdf).
