# Supplementary material for: Archaeorhizomycetes Spatial Distribution in Soils Along Wide Elevational and Environmental Gradients Reveal Co-abundance Patterns With Other Fungal Saprobes and Potential Weathering Capacities
Source: Front Microbiol. 2019 Apr 4;10:656. doi: 10.3389/fmicb.2019.00656 (PMC6458284; doi:10.3389/fmicb.2019.00656)
Supplement: Supplementary file 1 [file Data_Sheet_1.pdf]

Supplementary Material of the *Frontiers in Microbiology* paper:

Archaeorhizomycetes spatial distribution in soils along wide elevational and environmental gradients reveals co-abundance patterns with other fungal saprobes and potential weathering capacities

Eric Alejandro Pinto-Figueroa[1], Emily Seddon[1], Erika Yashiro[2], Aline Buri[3], Hélène Niculita-Hirzel[4†], Jan Roelof van der Meer[2†] and Antoine Guisan[1,3†*]

[1] Department of Ecology and Evolution, University of Lausanne, Switzerland

[2] Department of Fundamental Microbiology, University of Lausanne, Switzerland

[3] Institute of Earth Surface Dynamics, University of Lausanne, Switzerland

[4] Institute for Work and Health, University of Lausanne and Geneva, Switzerland

† Co-last authorship**;** * Correspondence: antoine.guisan@unil.ch

Content

1 Supplementary Figures and Tables

1.1 Supplementary Figures
- **Figure S1.** *In silico* PCR and coverage of diverse forward fungal ITS primers
- **Figure S2**. PCR amplification and HiSeq Illumina sequencing strategy
- **Figure S3.** Differences in the ITS1 region length of Archaeorhizomycetes sequences
- **Figure S4**. Hybrid open-reference OTUs procedure
- **Figure S5**. Procrustes analyses comparing NMDS with de novo versus referenced OTUs
- **Figure S6**. GLM diagnostic plots of Archaeorhizomycetes abundance
- **Figure S7**. GLM diagnostic plots of log-transformed Archaeorhizomycetes abundance
- **Figure S8.** Histograms of Archaeorhizomycetes sequences abundance at genus level
- **Figure S9**. Rarefaction curves of unique referenced and de novo OTUs at 97% identity
- **Figure S10**. Clusters of fungal ITS1 sequence reads at phyla level
- **Figure S11.** Rank abundance curve of referenced fungal OTUs.

1.2 Supplementary Tables
- **Table S1**. Full list of initial edaphic parameters.
- **Table S2**. Forward ITS1F barcode primers developed for demultiplexing with Illumina.
- **Table S3**. Reverse ITS2 barcode primers developed for demultiplexing with Illumina.
- **Table S4**. Topo-climatic variables used, at a 25 m resolution.
- **Table S5**. Summary of sequencing reads after rarefaction across the 103 sampling sites.
- **Table S6**. Selected fungal genera based on Spearman correlations.
- **Table S7**. Selected plant species based on correlations with Archaeorhizomycetes.
- **Table S8**. Selected edaphic factors based on correlations with Archaeorhizomycetes.
- **Table S9**. GLMs of Archaeorhizomycetes as a function of edaphic and climatic variables.

1.3 Supplementary References

1. Supplementary Figures and Tables

1.1 Supplementary Figures

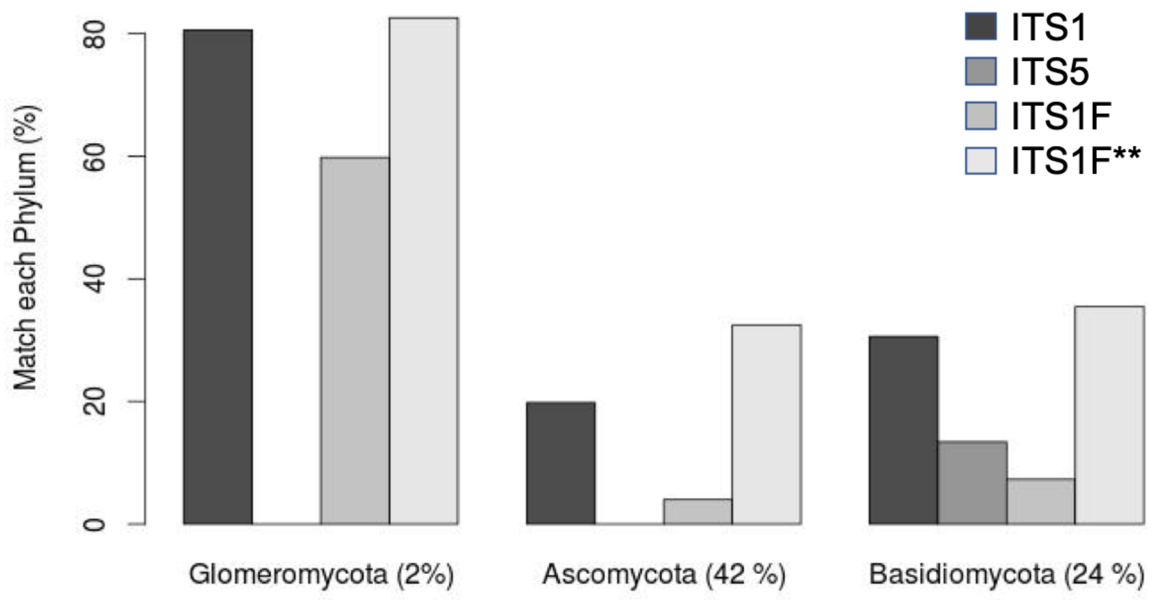

Supplementary Figure S1. *In silico* PCR using different forward fungal ITS primers on UNITE (12.11) dataset. The recovery percentage at phylum level shows that ITS1F** (Schmidt et al., 2013a) used in this work exhibit the lowest bias.

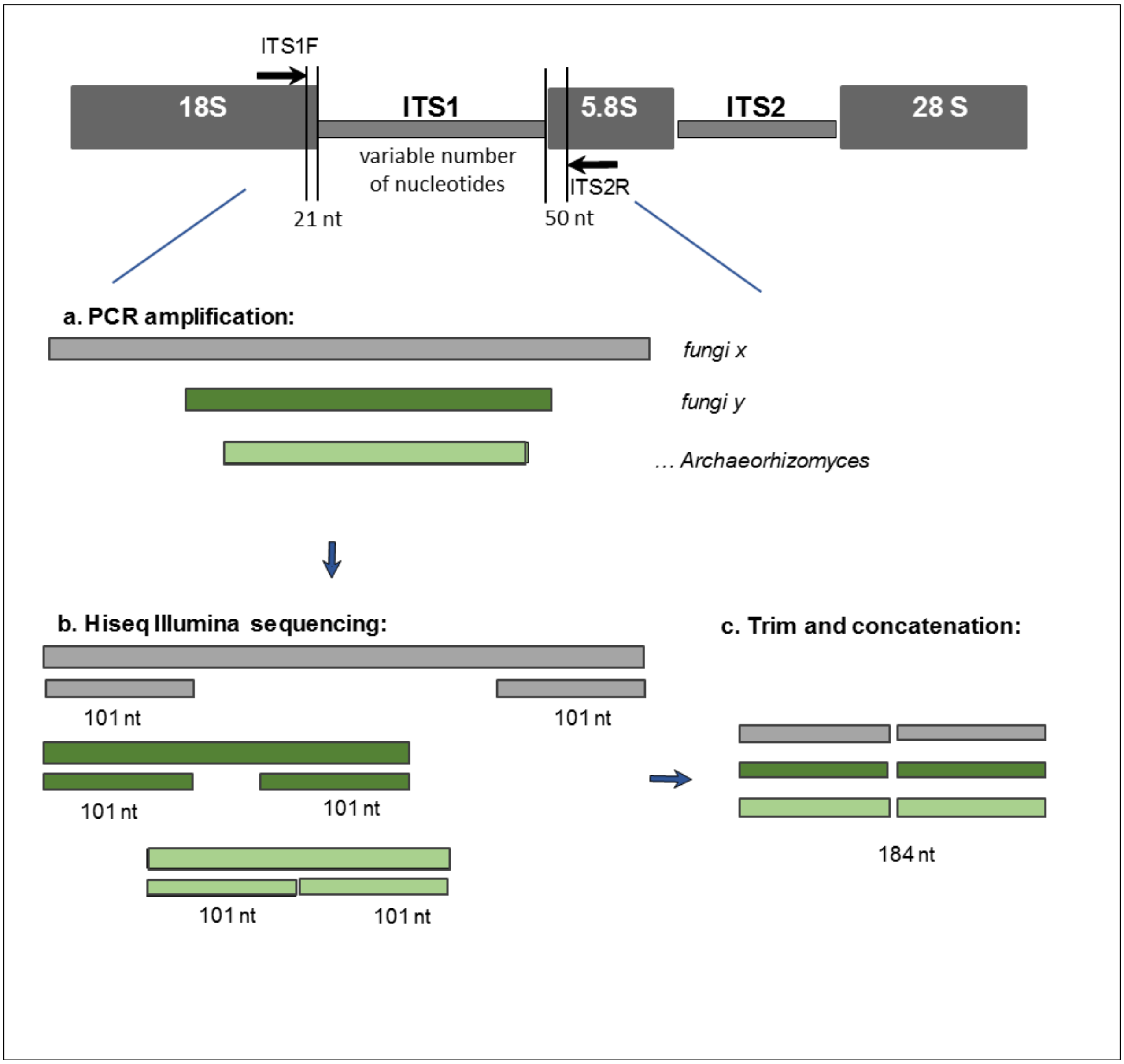

Supplementary Figure S2. . PCR amplification and HiSeq Illumina sequencing strategy. a) ITS1 amplicons ranging between 180 to 750 nucleotides (nt) were obtained by using the primer pairs ITS1F and ITS2R. b) HiSeq Illumina sequencing forward and reverse produce fastq sequences of 101 nt. c) custom scripts produce paired-end sequences of 92 nt per single read (total 184 nt). The Archaeorhizomyces amplicon size is expected to be around 183 – 192 nt.

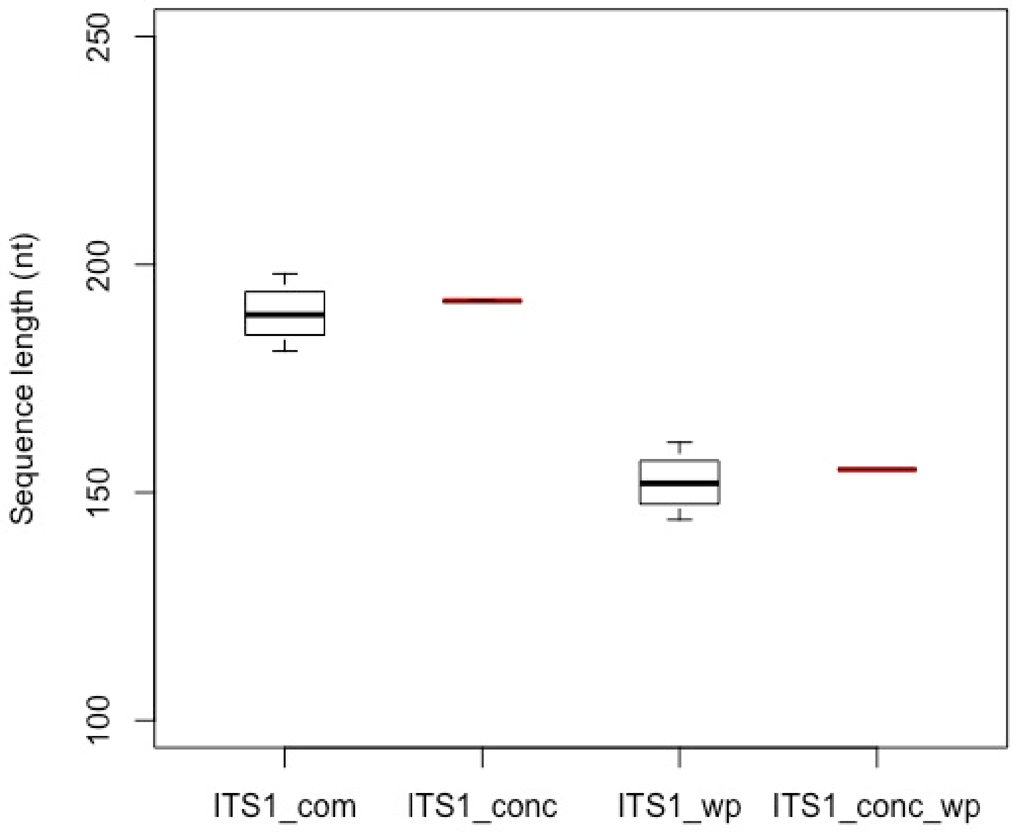

Supplementary Figure S3. Differences in the ITS1 region length of Archaeorhizomycetes sequences (UNITE-QIIME, 12.11). Complete (ITS1_com, median=189 nt) and concatenated (ITS1_conc, 192 nt) sequences including the forward ITS1F (Schmidt et al., 2013) and reverse ITS2 primers (White et al., 1990). Complete ITS1 without primers (ITS1_wp, median=152 nt) and concatenated ITS1 without primers (ITS1_conc_wp, 155 nt).

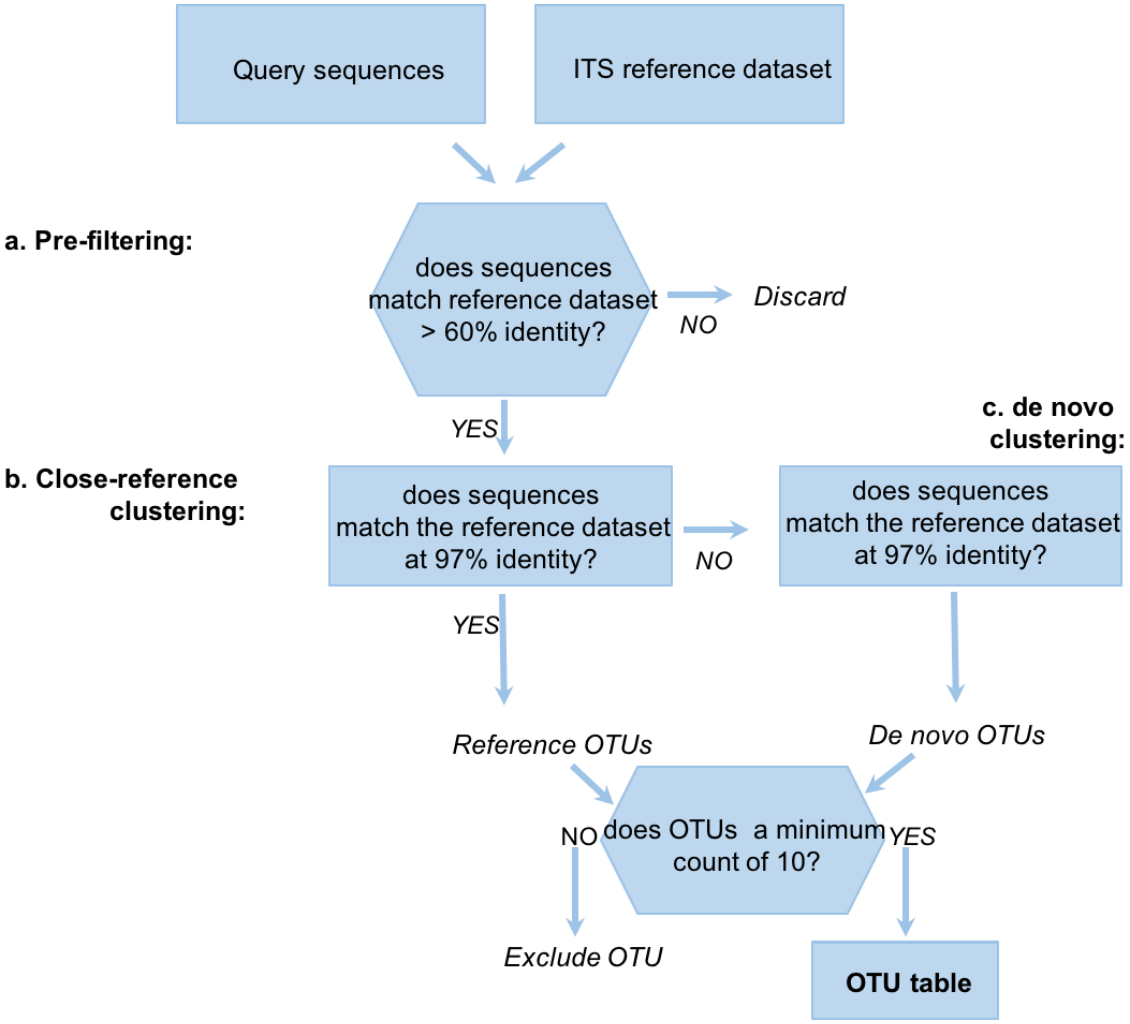

Supplementary Figure S4. Hybrid open-reference OTUs procedure (modified from Rideout et al., 2014). The ITS reference dataset was obtained from the QIIME-UNITE version 11.12.

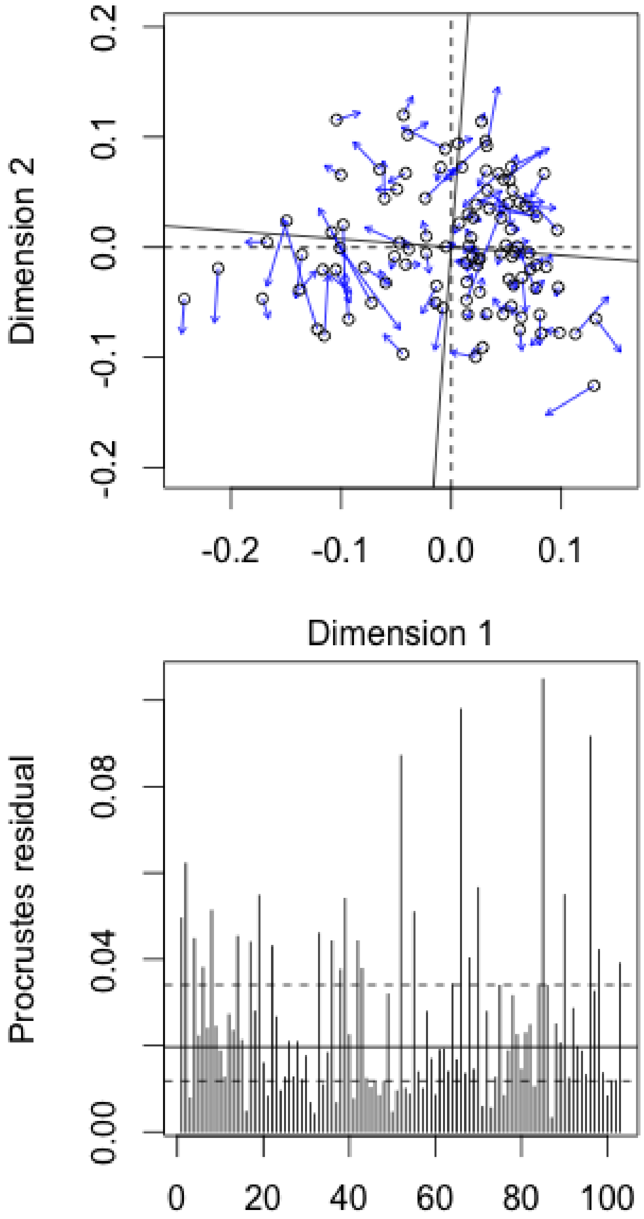

a)

b)

Supplementary Figure S5. Procrustes analyses (see Thioulouse et al., 2018) comparing non-metric multidimensional scaling (NMDS, with Bray-Curtis dissimilarity; Kruskal and Wish, 1978) topologies between de novo and referenced OTUs in QIIME 1.8 (Caporaso et al., 2010).

(A)

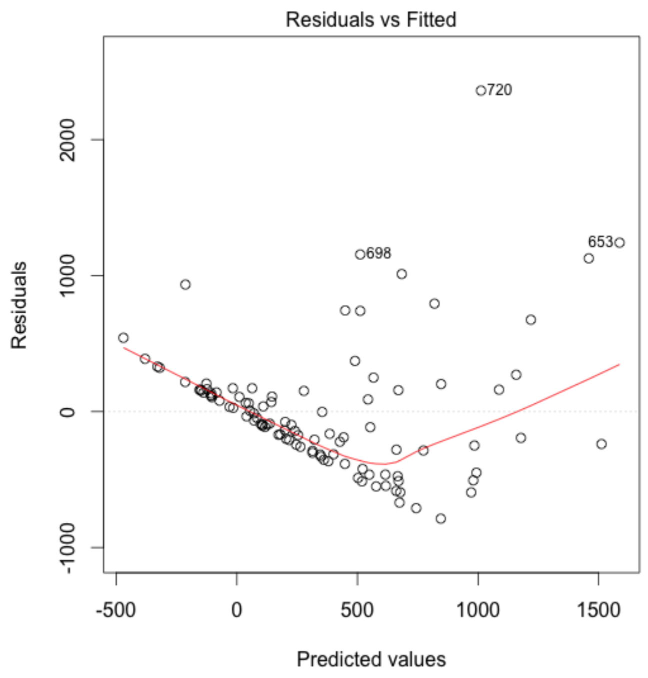

(B)

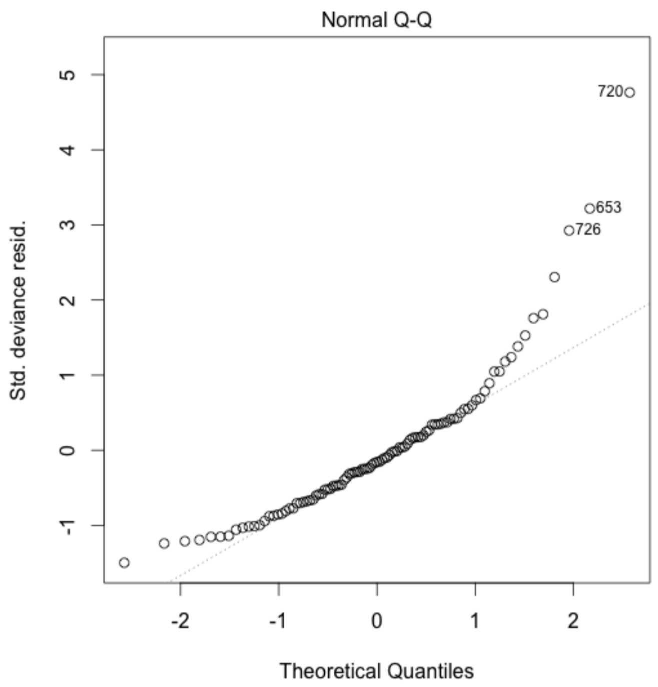

Supplementary Figure S6. GLM diagnostic plots of Archaeorhizomycetes abundance. In the residuals vs. predicted plot **(A)**, the larger residual fitted values at the upper left indicates heteroscedasticity (violation of homogeneity of variance). In the normal QQ-plot **(B)**, the residuals are not following the normal distribution.

(A)

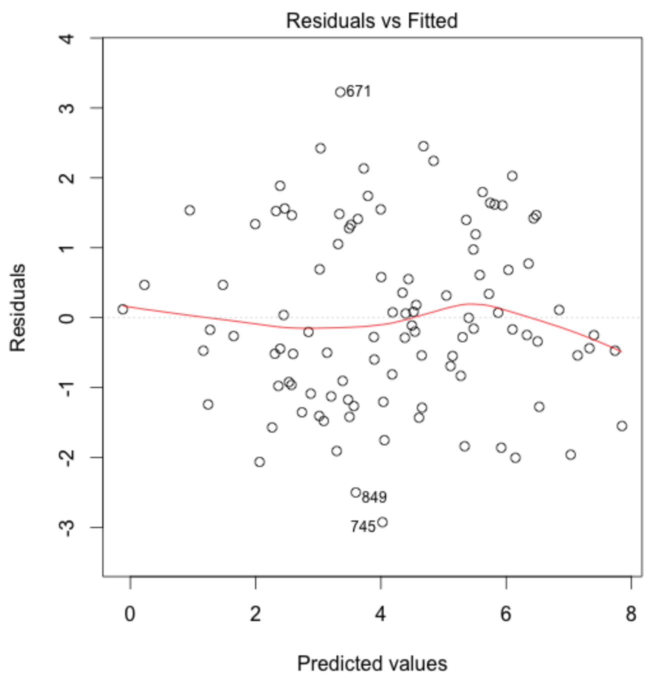

(B)

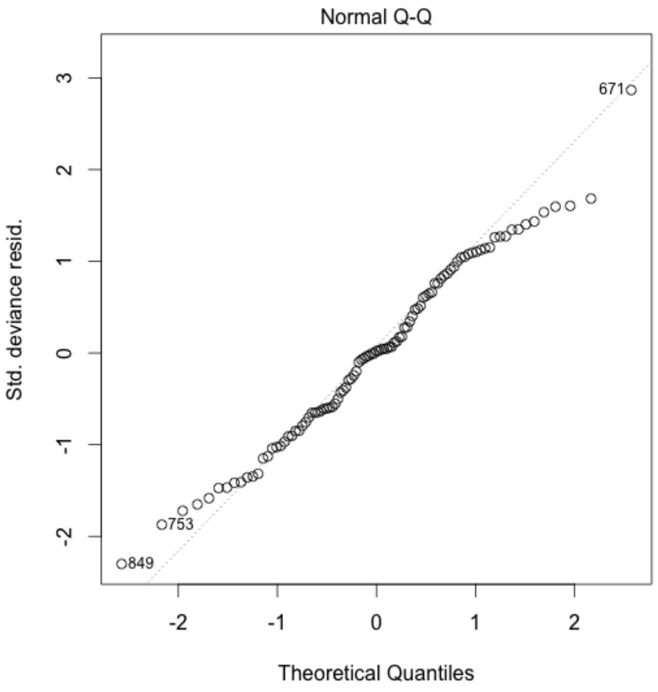

Supplementary Figure S7. GLM diagnostic plots of Archaeorhizomycetes abundance with log transformation. In the residuals vs. predicted plot **(A)**, the quasi-equal residual fitted values indicates homoscedasticity, homogeneity of variance. In the normal QQ-plot **(B)**, most of the residuals follow the normal distribution

(A)

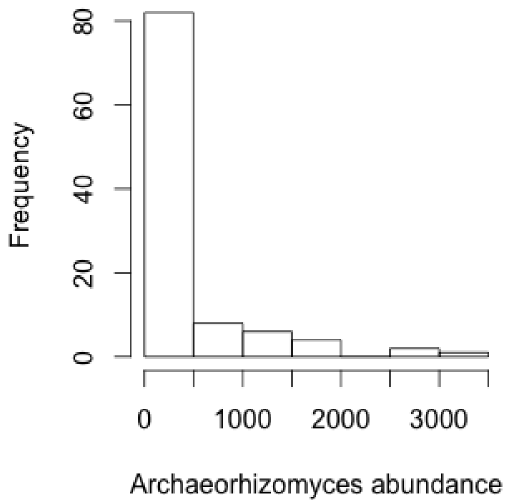

(B)

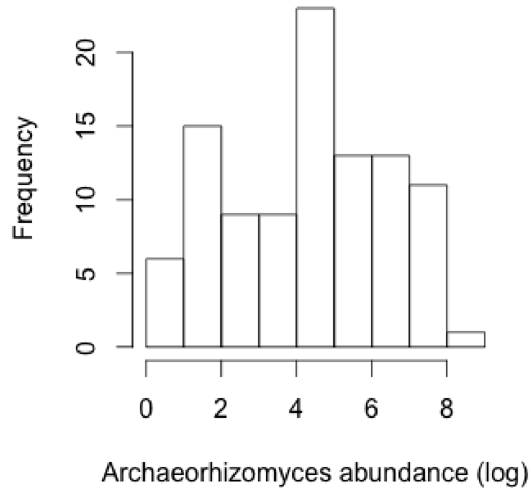

Supplementary Figure S8. Histograms of the abundance of Archaeorhizomycetes sequences at genus level (Archaeorhizomyces), **(A)** without logarithmic transformation and in **(B)** with logarithmic transformation.

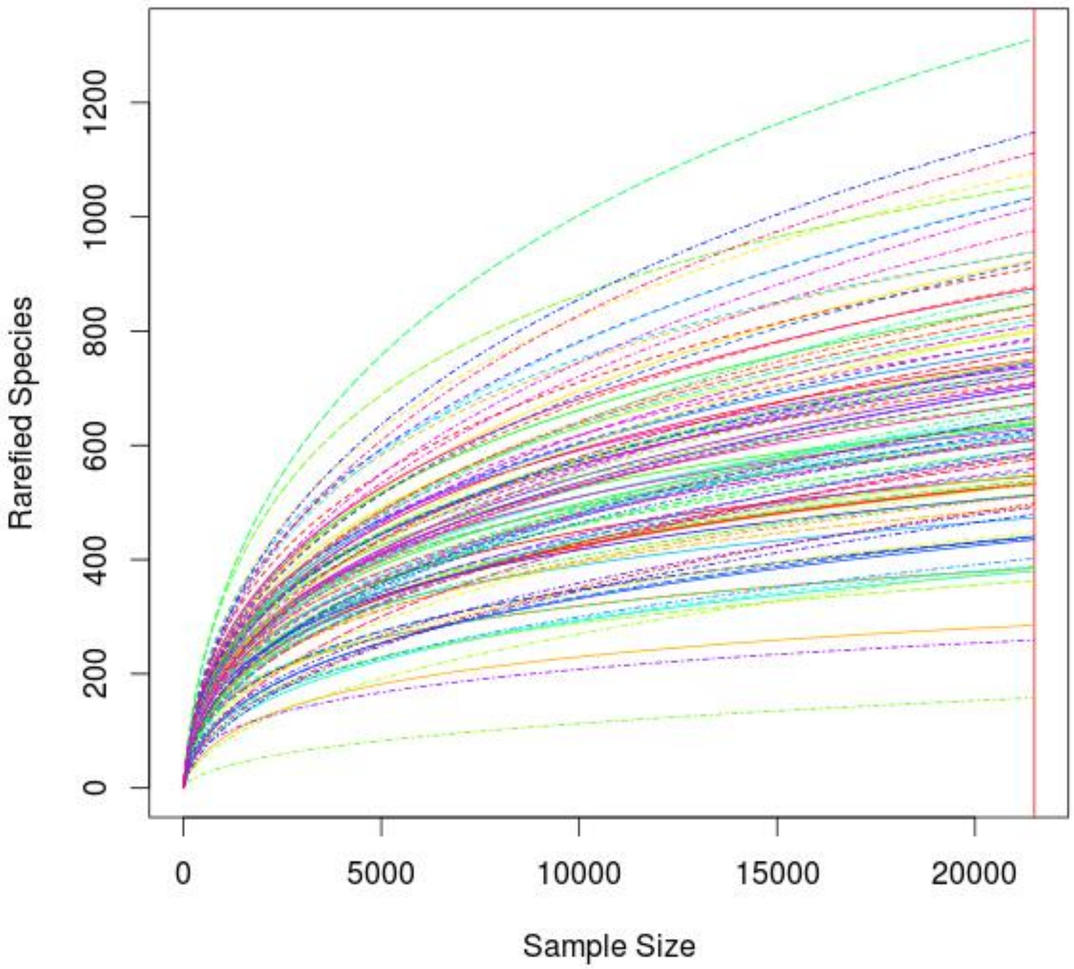

Supplementary Figure S9. Rarefaction curves showing the number of unique operational taxonomic units (OTUs, both referenced and *de novo*) at 97% identity among the 103 sampling sites as a function of the number of reads. Curves correspond to single sampling sites. The vertical line (in red) indicates the rarefaction point at 21,500 reads

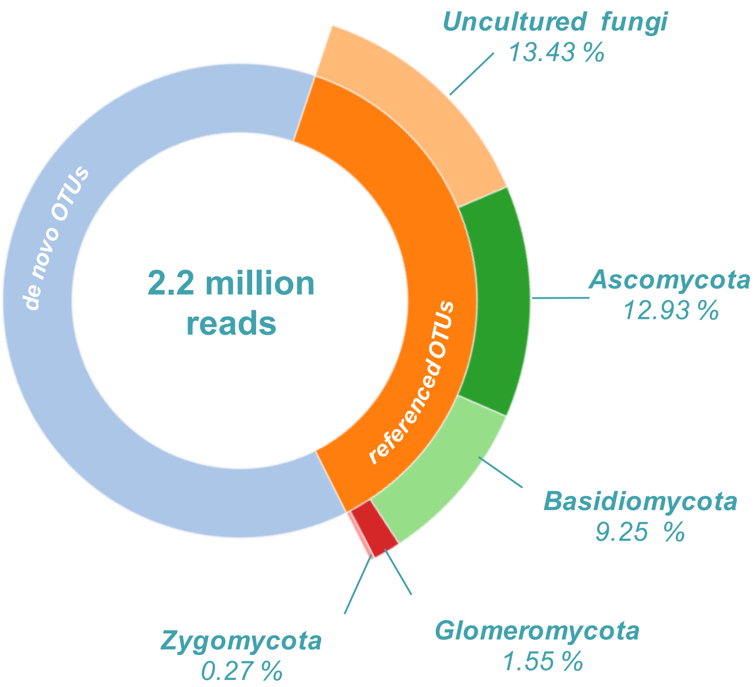

Supplementary Figure S10. Fungal ITS1 sequence reads across all sites, separated in *de novo* and referenced clusters, and attributed to phyla level.

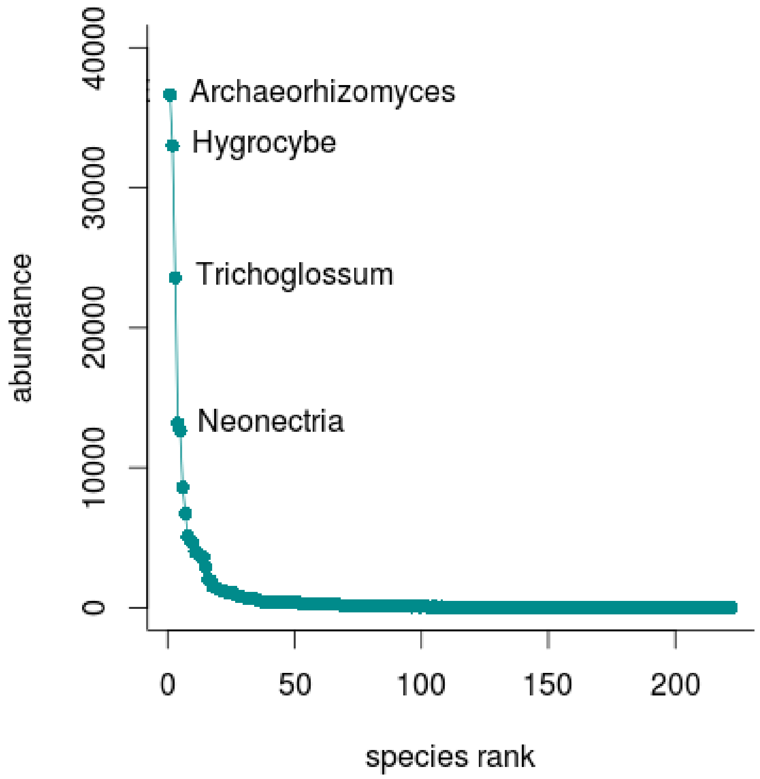

Supplementary Figure 11. Rank abundance curve for referenced OTUs (genus level) across the studied mountain region. The four most abundant fungal genera are shown.

1.2 Supplementary Tables

Supplementary Table S1. Full list of initial edaphic parameters (see Yashiro et al., 2016).

Category	Edaphic (soil) parameters	Abbreviation	Unit
General Physicochemical	Soil temperature at –5 cm	Soil temp	(°C)
	Bulk soil water content at 40°C	Water content	(wt %)
	Soil pH	Soil pH	Log (H)
	Electrical conductivity	EC 1.1	(1:1 microS cm^{-1})
	Total phosphorous content	P	(mg g^{-1})
	Soluble phosphorous content	Soluble P	(mg kg DW^{-1})
	Total organic carbon content	TOC	(wt %)
	Bulk C:N ratio	C:N	-
	Mineral carbon	MINC	(wt %)
	Organic matter content	OM	(wt %)
	Hydrogen index	HI	(mg g^{-1} TOC)
	Oxygen index	OI	(mg CO_2 g^{-1} TOC)
	Bulk nitrogen content	N	(wt %)
	Bulk carbon content	C	(wt %)
	Bulk hydrogen content	H	(wt %)
Parent Material	Phyllosilicates	Phyllosilicates	(%)
	Quartz	Quartz	(%)
	Feldspath-K	Feldspath	(%)
	Plagioclase-Na	Plagioclase	(%)
	Calcite	Calcite	(%)
	Goethite	Goethite	(%)
	Ankerite	Ankerite	(%)
	Indoses	Indoses	(%)
	Silicon dioxide	SiO_2	(wt %)
	Titanium dioxide	TiO_2	(wt %)
	Aluminum oxide	Al_2O_3	(wt %)
	Ferric oxide	Fe_2O_3	(wt %)
	Manganese oxide	MnO	(wt %)

**Mineral**	Magnesium oxide	MgO	(wt %)
	Calcium oxide	CaO	(wt %)
	Sodium oxide	Na_2O	(wt %)
	Potassium oxide	K_2O	(wt %)
	Diphosphorus pentoxide	P_2O_5	(wt %)
	Chromium oxide	Cr_2O_3	(wt %)
	Nickel oxide	NiO	(wt %)
**Isotopic**	Nitrogen stable isotope ratio	$d^{15}N$	(per mL vs. Air-N_2)
	Carbon stable isotope ratio	$d^{13}C$	(per mL V-PDB)

Supplementary Table S2. List of forward ITS1F barcode primers developed for demultiplexing with Illumina.

Name	Barcode	Forward_ITS1E
1_ITS1F	TGTAGG	TGTAGGTAAGTTTCADHCTTGC
2_ITS1F	CGT	CGTNNGAACCWGCGGARGGATC
3_ITS1F	GGA	GGANNGAACCWGCGGARGGATC
4_ITS1F	AGTC	AGTCNNGAACCWGCGGARGGATC
5_ITS1F	TGCT	TGCTNNGAACCWGCGGARGGATC
6_ITS1F	CACT	CACTNNGAACCWGCGGARGGATC
7_ITS1F	GACA	GACANNGAACCWGCGGARGGATC
8_ITS1F	ACCTAA	ACCTAANNGAACCWGCGGARGGATC
9_ITS1F	TTCCAA	TTCCAANNGAACCWGCGGARGGATC
10_ITS1F	TATTCC	TATTCCNNGAACCWGCGGARGGATC
11_ITS1F	CTTACC	CTTACCNNGAACCWGCGGARGGATC
12_ITS1F	TTCTTC	TTCTTCNNGAACCWGCGGARGGATC
13_ITS1F	ACCACG	ACCACGNNGAACCWGCGGARGGATC
14_ITS1F	AATGGT	AATGGTNNGAACCWGCGGARGGATC
15_ITS1F	ATACCT	ATACCTNNGAACCWGCGGARGGATC
16_ITS1F	TGGATT	TGGATTNNGAACCWGCGGARGGATC
17_ITS1F	CCTGTT	CCTGTTNNGAACCWGCGGARGGATC
18_ITS1F	CGTTAT	CGTTATNNGAACCWGCGGARGGATC
19_ITS1F	CGACTT	CGACTTNNGAACCWGCGGARGGATC
20_ITS1F	GTCCTT	GTCCTTNNGAACCWGCGGARGGATC
21_ITS1F	TCTCCT	TCTCCTNNGAACCWGCGGARGGATC
22_ITS1F	GGTAGT	GGTAGTNNGAACCWGCGGARGGATC
23_ITS1F	TTGTGT	TTGTGTNNGAACCWGCGGARGGATC
24_ITS1F	GGTGAC	GGTGACNNGAACCWGCGGARGGATC

Supplementary Table S3. List of reverse ITS2 barcode primers developed for demultiplexing with Illumina.

Name	Barcode	Reverse_ITS2E
1_ITS2R	TATTCG	TATTCGNNGCTGCGTTCTTCATCGATGC
2_ITS2R	TTCAAC	TTCAACNNGCTGCGTTCTTCATCGATGC
3_ITS2R	TCACCG	TCACCGNNGCTGCGTTCTTCATCGATGC
4_ITS2R	CCGTTC	CCGTTCNNGCTGCGTTCTTCATCGATGC
5_ITS2R	TTCTTG	TTCTTGNNGCTGCGTTCTTCATCGATGC
6_ITS2R	TGGCTT	TGGCTTNNGCTGCGTTCTTCATCGATGC
7_ITS2R	GCCTTA	GCCTTANNGCTGCGTTCTTCATCGATGC
8_ITS2R	ATTCTC	ATTCTCNNGCTGCGTTCTTCATCGATGC
9_ITS2R	CAGATT	CAGATTNNGCTGCGTTCTTCATCGATGC
10_ITS2R	GCTATT	GCTATTNNGCTGCGTTCTTCATCGATGC
11_ITS2R	CTCGCT	CTCGCTNNGCTGCGTTCTTCATCGATGC
12_ITS2R	TCTGAT	TCTGATNNGCTGCGTTCTTCATCGATGC
13_ITS2R	ATCTGC	ATCTGCNNGCTGCGTTCTTCATCGATGC
14_ITS2R	TAC	TACNNGCTGCGTTCTTCATCGATGC
15_ITS2R	TTG	TTGNNGCTGCGTTCTTCATCGATGC
16_ITS2R	AAG	AAGNNGCTGCGTTCTTCATCGATGC
17_ITS2R	CGT	CGTNNGCTGCGTTCTTCATCGATGC
18_ITS2R	GGA	GGANNGCTGCGTTCTTCATCGATGC
19_ITS2R	CAT	CATNNGCTGCGTTCTTCATCGATGC
20_ITS2R	AACC	AACCNNGCTGCGTTCTTCATCGATGC
21_ITS2R	CGTT	CGTTNNGCTGCGTTCTTCATCGATGC
22_ITS2R	TGCT	TGCTNNGCTGCGTTCTTCATCGATGC
23_ITS2R	CACT	CACTNNGCTGCGTTCTTCATCGATGC
24_ITS2R	GACA	GACANNGCTGCGTTCTTCATCGATGC

Supplementary Table S4. List of the climatic variables derived from the digital elevation model (DEM) and climatic measurements at a 25 m resolution (derived from base maps for topography, temperature, and precipitation; see https://www.unil.ch/ecospat/home/menuguid/ecospat-resources/data.html).

**Climatic parameter**	**Abbreviation**	**Unit**
Potential evapotranspiration	etp	mm/day
Maximum monthly average temperature	tmax	°C
Minimum monthly average temperature	tmin	°C
Minimum precipitation days/ growing season	pmax	pmin, days
Sum precipitation days/ growing season	psum	psum, days
Minimum precipitation days/ growing season	pmax	pmin, days
Growing degree-days	gdd	°C-day

Supplementary Table S5. Summary of sequencing reads after rarefaction across the 103 sampling sites.

Site ID	counts	Site ID	counts	Site ID	counts	Site ID	counts
**606**	22521	**679**	52867	**763**	613912	**850**	88552
**608**	208414	**689**	488643	**765**	1131633	**854**	625040
**618**	80292	**693**	155790	**769**	320662	**855**	603731
**619**	293957	**694**	28611	**770**	1058391	**856**	768777
**622**	169037	**698**	497351	**777**	118728	**858**	271711
**624**	504513	**699**	1019774	**779**	321138	**859**	147133
**625**	597757	**700**	219409	**780**	226049	**860**	379487
**628**	100325	**703**	453922	**789**	52081	**861**	574851
**631**	875435	**705**	415393	**791**	287679	**862**	515076
**633**	155327	**709**	393520	**800**	195080	**864**	197921
**643**	505243	**715**	490843	**803**	27273	**866**	848535
**644**	88473	**717**	934949	**805**	72351	**868**	84413
**649**	746128	**720**	193432	**806**	619439	**870**	475797
**652**	197104	**722**	289584	**812**	30100	**871**	52829
**653**	113024	**724**	666894	**814**	1105290	**872**	1058027
**655**	103915	**725**	583389	**818**	285270	**873**	180568
**658**	21710	**726**	164625	**825**	572841	**881**	95680
**659**	1091220	**737**	896272	**831**	318835	**884**	121843
**661**	53273	**738**	456725	**833**	790270	**885**	395397
**666**	439864	**744**	754911	**835**	609902	**887**	616614
**669**	1070886	**745**	174124	**841**	390451	**890**	202483
**670**	64713	**746**	643944	**842**	849298	**894**	695878
**671**	178989	**748**	215072	**843**	716307	**899**	115315
**676**	443884	**753**	431620	**844**	477106	**904**	265606
**677**	686295	**754**	583288	**847**	711575	**909**	699612
**678**	157517	**757**	736467	**849**	494549		

Supplementary Table S6. Selected fungal genera based on Spearman correlations. Six different fungal genera were selected from a total of 220. The Genbank accession numbers of the fungal species (specific OTUs 97% identity) and relevant information for each fungus are provided.

Spearman correlation	Genus	Species	Genbank	Feature	Others
0.337	Thypula	*Typhula ishikariensis*	AF193350	cold soil	plant saprotrophic and pathogenic during snow cover
0.313	Exophiala	*Exophiala sp.*	AB488490	soil	saprotrophic & potential pathogen
		Exophiala salmonis	AM176667	water	opportunistic pathogen
0.306	Cephalosporium (taxon synonymous Acremonium)	*Acremonium alcalophilum*	AB540579	soil	saprotrophic
		Acremonium rutilum	AB540580	soil	saprotrophic
		Acremonium psammosporum	GU566287	soil	saprotrophic
- 0.288	Omphalotus	*Omphalotus illudens*	AY313271	soil	decaying stumps, buried roots
- 0.294	Suillus	*Suillus sp.*	GU187544	soil	ectomycorrhizal
		Suillus sp.	GU553371	soil	ectomycorrhizal
		Suillus sp.	L54082	soil	ectomycorrhizal
		Suillus sp.	L54112	soil	ectomycorrhizal
- 0.325	Tubeufia	*Tubeufia helicomyces*	AY916461	soil	decaying grass stems in damp situations

Supplementary Table S7. Selected plant species based on Spearman correlation with Archaeorhizomycetes. The plant species were reduced from 448 species to six plant species. The families for each plant species and relevant features are provided.

Spearman correlation	Family	Species	Feature	Others
0.391	Cyperaceae	*Carex flacca*	widespread	Non-mycorrhizal / drought resistant
0.327	Cistaceae	*Helianthemum nummularium*		Mycorrhizal / drought resistant
0.325	Rosaceae	*Alchemilla coriacea*	cold soil	Weakly mycorrhizal
0.318	Fabaceae	*Anthyllis vulneraria*		Mycorrhizal fungi protecting erosion
0.313	Asteraceae	*Leucanthemum vulgare*		Endophyte interactions
0.290	Polygonaceae	*Polygonum viviparum*	widespread	Ectomycorrhizal

Supplementary Table S8. The 10 edaphic factors selected based on Spearman correlation, from all available factors.

Edaphic parameters	Abbreviation	Unit
Total organic carbon content	TOC	(wt %)
Bulk nitrogen content	N	(wt %)
Bulk carbon content	C	(wt %)
Bulk hydrogen content	H	(wt %)
Phyllosilicates	Phyllosilicates	(%)
Quartz	Quartz	(%)
SiO_2	SiO_2	(wt %)
Al_2O_3	Al_2O_3	(wt %)
MgO	MgO	(wt %)
K_2O	K_2O	(wt %)

Supplementary Table S9. Full list of generalized linear model (GLM) and generalized quadratic model results of Archaeorhizomycetes, edaphic and climatic variables. Generalized quadratic model results are indicated with a squared superscript. Levels of significance were evaluated by Benjamini- Hochberg (BH) multiple correction (* *p*-value < 0.05, ***p*-value < 0.01 and *** *p*-value < 0.001).

Type	Variable	Estimate	Standard error	t-value	p-value	BH
**Edaphic**	Nitrogen	-15.8727	4.768	-3.329	0.0014**	0.0041
	Hydrogen	8.2439	2.6333	3.1306	0.0025**	0.006
	Phyllosilicates	-0.1663	0.0632	-2.6302	0.0104*	0.0208
	SiO_2	0.1925	0.0917	2.0981	0.0393	**0.059**
	Al_2O_3	0.8353	0.3383	2.4694	0.0158*	0.0272
	MgO	0.6906	0.3953	1.747	0.0848	0.0969
	OM	0.4077	0.2141	1.9043	0.0608	0.0782
**Climatic**	tmin	0.0137	0.0066	2.0619	0.0427	**0.0603**
	gdd	0.018	0.0051	3.5588	0.0007**	0.0026
	pmin	0.0464	0.0345	1.3443	0.1829	0.1898
	psum	-0.037	0.0088	-4.2105	0.0001***	0.0012
	etp	-0.2312	0.0966	-2.3925	0.0193*	0.0308
**Edaphic**	TOC^2	-0.0818	0.0266	-3.0769	0.0029**	0.0064
	$Nitrogen^2$	10.3445	3.089	3.3488	0.0013**	0.0041
	$Carbon^2$	0.1119	0.0312	3.5885	0.0006**	0.0026
	$Hydrogen^2$	-3.2331	0.9003	-3.5913	0.0006**	0.0026
	$Phyllosilicates^2$	0.0033	0.001	3.2703	0.0016**	0.0044
	${Al_2O_3}^2$	-0.0272	0.0143	-1.896	0.0619	0.0782
	OM^2	-0.006	0.0032	-1.8534	0.0678	0.0814
**Climatic**	$tmin^2$	0	0	2.5483	0.0129*	0.0238
	gdd^2	0	0	-4.1216	0.0001***	0.0012
	$pmax^2$	-0.0002	0.0001	-1.3234	0.1898	0.1898
	$psum^2$	0	0	3.6823	0.0004***	0.0026
	etp^2	0.0013	0.0008	1.5705	0.1206	0.1315

1.3 Supplementary references

Caporaso, J. G., Kuczynski, J., Stombaugh, J., Bittinger, K., Bushman, F. D., Costello, E. K., et al. (2010). QIIME allows analysis of high- throughput community sequencing data. *Nat. Methods* 7, 335–336. doi:10.1038/nmeth0510-335.

Kruskal, J., and Wish, M. (1978). *Quantitative Applications in the Social Sciences: Multidimensional scaling.* Thousand Oaks, California: SAGE Publications, Inc. doi:10.4135/9781412985130.

Rideout, J. R., He, Y., Navas-Molina, J., Walters, W., Ursell, L. K., Gibbons, S. M., et al. (2014). Subsampled open-reference clustering creates consistent, comprehensive OTU definitions and scales to billions of sequences. *PeerJ* 2, e545. doi:10.7717/peerj.545.

Schmidt, P.-A., Bálint, M., Greshake, B., Bandow, C., Römbke, J., and Schmitt, I. (2013). Illumina metabarcoding of a soil fungal community. *Soil Biol. Biochem.* 65, 128–132. doi:10.1016/j.soilbio.2013.05.014.

Thioulouse, J., Dray, S., Dufour, A.-B., Siberchicot, A., Jombart, T., and Pavoine, S. (2018). *Multivariate analysis of ecological data with ade4*. New York, NY: Springer-Verlag doi:10.1007/978-1-4939-8850-1.

Yashiro, E., Pinto-Figueroa, E., Buri, A., Spangenberg, J. E., Adatte, T., Niculita-Hirzel, H., et al. (2016). Local environmental factors drive divergent grassland soil bacterial communities in the western Swiss Alps. *Appl. Environ. Microbiol.* 82, 6303–6316. doi:10.1128/AEM.01170-16.
